# Supplementary material for: Non-Steroidal Estrogens Inhibit Influenza Virus by Interacting with Hemagglutinin and Preventing Viral Fusion
Source: Int J Mol Sci. 2023 Oct 19;24(20):15382. doi: 10.3390/ijms242015382 (PMC10607366; doi:10.3390/ijms242015382)
Supplement: Supplementary file 1 [file ijms-24-15382-s001.zip › ijms-2665367-supplementary.pdf]

Supplementary Data

Non-Steroidal Estrogens Inhibit Influenza Virus by Interacting with Hemagglutinin and Preventing Viral Fusion

Elisa Franzi [1], Gregory Mathez [1], Soraya Dinant [2], Charlotte Deloizy [2], Laurent Kaiser [3,4], Caroline Tapparel [5], Ronan Le Goffic [2], and Valeria Cagno [1,*]

[1] Institute of Microbiology, Lausanne University Hospital, University of Lausanne, 1011 Lausanne, Switzerland

[2] INRAE, UVSQ, UMR892 VIM, Université Paris-Saclay, 78350 Jouy-en-Josas, France

[3] Laboratory of Virology, Division of Infectious Diseases and Division of Laboratory Medicine, University Hospitals of Geneva, University of Geneva, 1206 Geneva, Switzerland

[4] Center for Emerging Viruses, Geneva University Hospitals, 1205 Geneva, Switzerland

[5] Department of Microbiology and Molecular Medicine, University of Geneva, 1206 Geneva, Switzerland

* To whom correspondence should be addressed.

Valeria Cagno

Institute of Microbiology of Lausanne

Rue du Bugnon 48

1011 Lausanne

+41213142611

valeria.cagno@chuv.ch

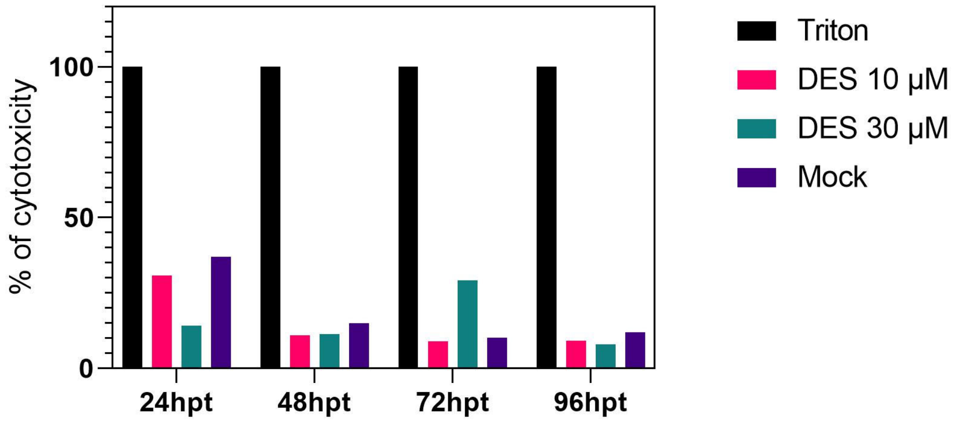

Figure S1. Viability of respiratory airways treated with DES**.** Mucilair tissues were treated apically every 48h with DES diluted in medium or with only medium (mock). LDH was quantified in apical washes and compared to the apical washes of tissues treated with Triton.

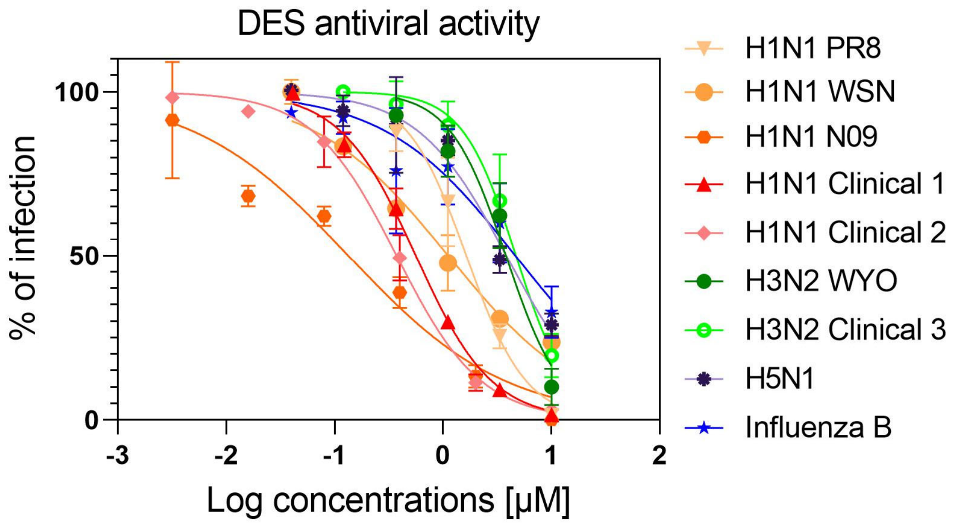

Figure S2. Dose response curves with DES and different strains of influenza virus. DES was added on MDCK cells or MDCK-Siat cells before and during infection with the different strains of influenza virus at MOI 0.01. Cells were fixed and subjected to immunostaining 24hpi. Infected cells were counted, and dose-response curves were built with GraphPad Prism. Results are mean and SEM of 2 to 3 independent experiments.

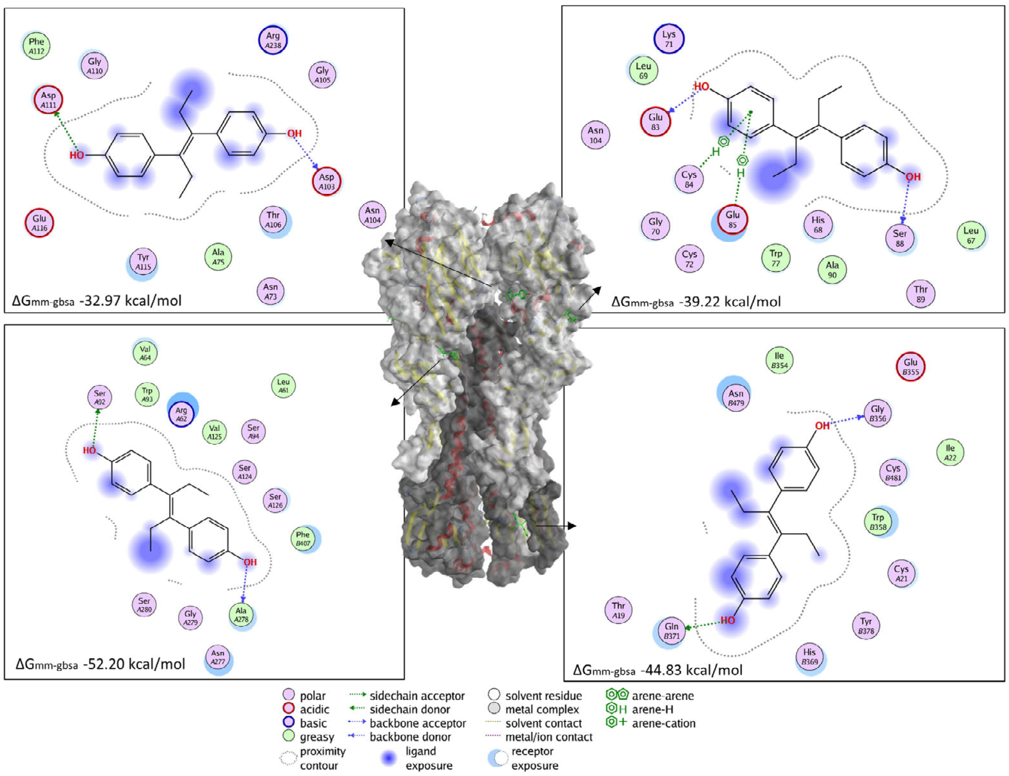

Figure S3. Interaction of DES on the H1 hemagglutinin. The four binding sites identified with SwissDock and re-docked with Maestro Schrödinger are represented with their position on the hemagglutinin trimer, their interaction map done with MOE 2019.0102 and their estimated $\Delta G_{mm\text{-}gbsa}$ after Prime MM-GBSA with Maestro Schrödinger.

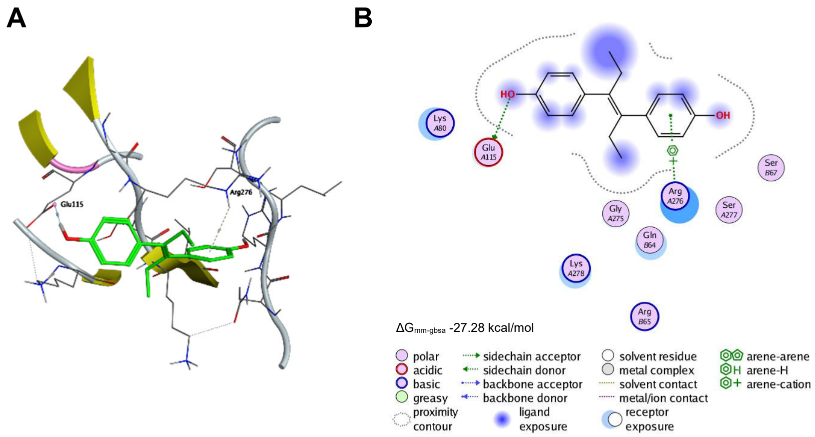

Figure S4. Interaction of DES on influenza B hemagglutinin. Representation of the binding (**A**) and interaction map (**B**) of DES on influenza B hemagglutinin with PDB 4M44. The corresponding binding site identified in H1 influenza A virus as the most favorable was taken to evaluate the binding of DES. The molecule was docked with Maestro Schrödinger and refined with Prime MM-GBSA. Visualization and interaction map were done with MOE 2019.0102. The estimated $\Delta G_{mm\text{-}gbsa}$ was performed with Prime MM-GBSA (Maestro Schrödinger).

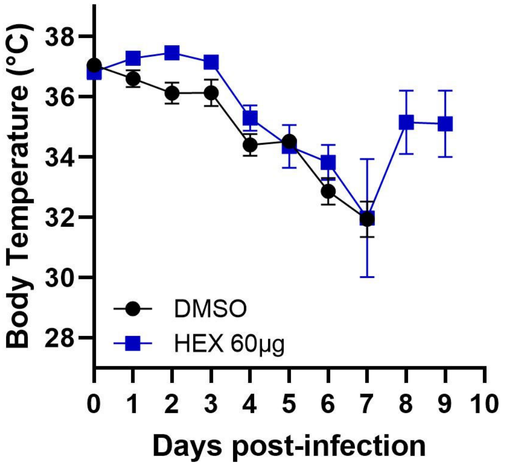

Figure S5. Hexestrol effect on body temperature. Mice were infected with 10 PFU of N09 and treated or not with hexestrol (60 µg/mouse). The temperature was measured daily rectally.
